# Discovery of the Anæsthetic Effects of Chloroform

**Published:** 1859-12

**Authors:** 


### DISCOVERY OF THE ANÆSTHETIC EFFECTS OF CHLOROFORM.

A writer in the Household Words gives the following description of the discovery of the powerful anæsthetic properties of chloroform:

To Professor Simpson, of Edinburgh, belongs the distinguished credit of introducing chloroform, which has nearly superceded all other anæsthetics. Possessed with the notion that something better than ether existed in the chemical world, the professor set about deliberately to examine any volatile substance which afforded a promise of revealing the required properties. Various gases and liquids were experimented upon; and at last chloroform—a ponderous liquid which provoked no great expectations; and only known as a chemical curiosity in the laboratory—was brought to the trial. Doctor Simpson, with his two assistants, sat down late one night, after an arduous day's toil, and when most physicians, as well as patients, were wrapped in sleep, began to inhale various substances which had been collected. A small bottle of chloroform had been raked up out of some obscure corner, and was to take its turn with the rest. Each experimenter provided himself with a tumbler or finger-glass, a portion of each selected fluid was poured into the bottom of it, and the glass was placed over warm water to favor evolution of vapor. Holding the mouth and nostrils over the vessel, these votaries of science courageously explored this *terra incognita* by inhaling one vapor after another. At last each charged his tumbler from the small bottle of chloroform, "when immediately," says Professor Miller, "an unwonted hilarity seized the party; they became bright-eyed and very happy, and conversed with such intelligence, as more than usually charmed other listeners who were not taking part in the proceedings. But suddenly there was a talk of sounds being heard like those of a cotton-mill, louder and louder; a moment more, then all was silent, and then—crash.

"On awaking, Dr. Simpson's first perception was mental: 'This is far stronger and better than ether,' he said to himself. His second was to note that he was prostrate on the floor, and that his friends were confused and alarmed. Hearing a noise, he turned round, and saw his assistant, Dr. Duncan, beneath a chair, his jaws dropped, his eyes staring and his head half bent under him; quite unconscious, and snoring in a determined and alarming manner. More noise still, and much motion, and then his eyes overtook Doctor Keith's feet and legs making various efforts to overturn the table, or more properly to annihilate every thing that was on it.

"All speedily regained their senses, and from that day, or rather from the middle of that night, dates the discovery of the marvelous properties of chloroform."

